# An HVOF-Sprayed (Cr_3_C_2_-NiCr+Co) Composite Coating on Ductile Cast Iron: Microstructure, Mechanical Properties, and Scratch Resistance

**DOI:** 10.3390/ma17071484

**Published:** 2024-03-25

**Authors:** Marzanna Ksiazek, Katarzyna Łyp-Wrońska

**Affiliations:** Department of Non-Ferrous Metals, AGH University of Krakow, al. A. Mickiewicza 30, 30-059 Krakow, Poland; klyp@agh.edu.pl

**Keywords:** HVOF, (Cr_3_C_2_-NiCr+Co) composite coating, mechanical properties, scratch bond strength, coating quality

## Abstract

High-velocity oxy-fuel (HVOF) thermally sprayed Cr_3_C_2_-NiCr coatings have been shown to be effective in shielding important machinery and equipment components from wear in harsh, high-temperature conditions. In this investigation, the HVOF thermal spray coating technique was used to deposit Cr_3_C_2_-NiCr powder with 10% Co particles onto ductile cast iron. The effect of the Co particles on the mechanical, tribological, and microstructure characteristics of a Cr_3_C_2_-NiCr/ductile cast iron system was investigated. The microstructure analysis employed various techniques, including light microscopy, X-ray diffraction (XRD), scanning electron microscopy (SEM), transmission electron microscopy (TEM), and energy-dispersive X-ray spectroscopy (EDS). Scratch tests were applied to analyze the coating quality and adhesion. The coatings created using the HVOF spray method with Cr_3_C_2_-NiCr powders mixed with Co particles exhibited a dense structure containing large Co particles, partially melted, and very fine Cr_3_C_2_ particles embedded into the NiCr alloy matrix. Additionally, they possessed high hardness and excellent adhesion to the substrate. The results of bending strength tests were also presented, together with information on the coating’s microhardness and fracture toughness. These included an analysis of the cracks and delamination in the Cr_3_C_2_-NiCr/ductile cast iron system. It was observed that the addition of Co particles significantly increased the resistance to cracking and wear behavior in the studied system.

## 1. Introduction

Various thermal spraying techniques, including but not limited to HVOF (high-velocity oxy-fuel), are widely used for coating applications to enhance protection against wear and the erosion and corrosion of large structural components. This is particularly relevant in industries such as thermal and nuclear power plants, where industrial turbine blades require effective protective coatings [1,2]. These techniques are also utilized in the regeneration of machine parts, involving comprehensive repair of elements such as the working surfaces of shafts, pump components, bushings, transport rollers, and guides [3,4]. In particular, the HVOF process is dedicated to applying coatings based on chromium and tungsten carbides on substrates made of iron, aluminum, and magnesium alloys [5,6]. The HVOF process offers numerous benefits. It is capable of ejecting partially molten particles at exceptionally high velocities, reaching speeds of around 900 m/s. This results in the formation of a dense coating that adheres effectively to the substrate. However, it is important to note that the specified velocity of 900 m/s may vary based on the process conditions and the type of equipment used. Additionally, the coatings exhibit a fine-grained microstructure, a low oxide content, and minimal carbide decomposition. As a result, the coatings produced using HVOF have a significantly higher hardness compared to those created using the conventional plasma spraying technique [7,8,9,10]. HVOF technology stands out for its direct applicability in real boiler installations. Unlike techniques such as CVD or PVD, it does not impose restrictions on the coating thickness or alter the substrate material’s structure. HVOF-sprayed coatings based on Cr_3_C_2_-NiCr are primarily utilized to protect against the erosive and corrosive wear of machine parts and devices operating at elevated temperatures, including temperatures up to 870 °C. This is attributed to their high structural stability under heavy loads for prolonged periods, excellent resistance to abrasive wear, and favorable sliding properties, characterized by low coefficients of friction within the temperature range of 25–850 °C [11,12]. Additionally, the hard and well-adhering layer of chromium oxide formed on their surface during the HVOF process, resulting from chromium’s direct reaction with oxygen, renders them resistant to oxidation in the temperature range of 650–1200 °C. This layer serves as an effective diffusion barrier during the process, preventing the material from losing its hardness and abrasion resistance [13,14]. However, it is widely acknowledged that at temperatures between 850 and 900 °C, further oxidation leads to the breakdown of the protective Cr_2_O_3_ oxide scale, which is crucial for their resistance to oxidation and corrosion, resulting in the formation of volatile CrO_3_. The coating’s corrosion resistance is effectively provided by the NiCr matrix; however, the carbide particles are the main component affecting its wear resistance. In Cr_3_C_2_-NiCr coatings produced using supersonic spraying, smaller chromium carbide particles, reaching nanometric sizes, have been found to enhance wear resistance and smoothness. However, due to the intensification of the decarburization process in the case of smaller chromium carbide particles, nanostructured coatings show a lower fracture toughness compared to conventional coatings. The increased microhardness of the coating is attributed to the dissolution of carbon in the metal matrix during the decarburization process. Nevertheless, this process can lead to the formation of brittle structures (Cr_7_C_3_, Cr_23_C_6_), which may adversely affect durability [15,16,17,18,19,20]. The great advantage of these coatings is the similar expansion coefficient of iron and chromium carbide (Cr_3_C_2_) [21,22], which has an impact on the minimization of internal stresses in the process of spraying these coatings onto substrates made of iron alloys. According to the studies [23,24,25], starting powders with a regulated morphology and grain size are used in an effort to enhance the mechanical, tribological, and microstructural characteristics of Cr_3_C_2_-NiCr HVOF coatings. In particular, the studies [26,27] have revealed that adding nickel particles to starting powder composed of chromium and tungsten carbide leads to decreased porosity and the formation of a beneficial structure, resulting in enhanced mechanical durability and wear resistance during the HVOF process. This enhancement is attributed to the increased plasticity of the matrix due to the doping. It has been effectively conveyed by Zhou et al. [18] and Varis et al. [19] that one of the main factors influencing the mechanical properties of coatings is the strengthening of the binder (NiCr). Also, the addition of Co particles to the starting powder base of Cr_3_C_2_-NiCr, owing to cobalt’s resistance to oxidation and thermal stability, is expected to reinforce the binder, enhancing its mechanical properties, adhesion, thermal stability, and oxidation resistance, thereby extending the coating’s lifespan in extremely severe conditions. It is also worth noting that the application of heat treatment to Cr_3_C_2_-NiCr coatings and their laser remelting have had a beneficial effect on their mechanical and tribological properties [28,29,30,31].

The aim of the work was to assess the effect of modifying the chemical composition by doping standard Cr_3_C_2_-25NiCr powders with metallic Co particles during the consolidation of coatings onto ductile cast iron in the HVOF process on the microstructure, mechanical properties, and scratch resistance of the coating system (Cr_3_C_2_-NiCr)/ductile cast iron, combined with an analysis of the cracking and delamination of the coating at the interface.

## 2. Materials and Methods

### 2.1. Preparation of the Coatings

Coatings of Cr_3_C_2_-NiCr and Cr_3_C_2_-NiCr+Co were applied using supersonic flame spraying of commercially available carbide powder containing Cr_3_C_2_-25(Ni20Cr) (75 wt% Cr_3_C_2_-25 wt.% NiCr) with a nominal grain size distribution of −45 + 5.5 µm (Diamalloy 3004 Sulzer Metco, Pfattikon, Switzerland) onto a ductile iron substrate. The Cr_3_C_2_-NiCr+Co composite coating was obtained by introducing 10 wt.% of 20 µm Co particles (Xi’an Function Material Group Co., Ltd., High-Tech Zone, Xi’an City, China) into the carbide powder. The volume composition of the powder mixture used to create the composite coating was as follows: 67.5 wt.% Cr_3_C_2_-22.5 wt.% NiCr-10 wt.% Co. A plasma equipment firm (Siemianowice, Silesia, Poland) employed the HV-50 HVOF spraying equipment to apply the coating. Table 1 summarizes the spraying parameters that were optimized. The substrate made of EN-GJS-500-7 ductile iron had the following chemical composition—3.61% C, 2.29% Si, 0.45% Mn, 0.045% P, 0.009% S, 0.03% Cr, 0.01% Ni, 0.057% Mg, 0.75% Cu, and the rest Fe (in weight percentages)—and was characterized by the following mechanical properties: yield strength = 340 (MPa), tensile strength = 500 (MPa), elongation = 7%, hardness = 220 HB. The substrate samples measured 100 by 15 by 5 mm^3^. For better coating mechanical adherence, the substrates’ surfaces were sandblasted using a loose corundum with 20-mesh granulation prior to spraying. The substrate’s surface roughness parameter R_a_ was 30 μm. The average thickness of the applied coating was 250 µm.

### 2.2. Microstructure Characterization

The microstructure and chemical composition of the coating/substrate system were examined using a Zeiss Axio Observer Zm1 light microscope (LM, Jena, Germany), a Scios DualBeam FEI scanning electron microscope (SEM, Valley City, ND, USA), and a JEOL 2010 ARD transmission electron microscope (TEM, Akishima, Japan) equipped with EDS spectrometers. For the TEM examinations, thin foils of the coating/substrate specimens were prepared using a Gatan PIPS 691 V3.1 ion thinner in Pleasanton, CA, USA, following standard procedures such as cutting out a 3 mm diameter disc, thinning using a dimpler, and ion polishing [32]. Phase composition studies were conducted using the X’Pert Pro Panalytical Diffractometer (Malvern Panalytical Ltd., Cambridge, UK) in the angular range of 20–90° with CuKα radiation (wavelength λ of 1.54 Å, X-ray power of 45 kV and 40 mA). The obtained spectra underwent preliminary numerical processing using the “EVA” software (Diffrac.Eva V4), involving background removal and noise reduction using Fourier transform. Phase identification was performed with the assistance of the ICDD database. Utilizing Rietveld analysis of the XRD data with GSAS/EXPGUI software (https://subversion.xray.aps.anl.gov/trac/EXPGUI accessed on 13 March 2024), a set of software phase compositions was derived, and the average crystallite size was calculated using the Scherrer formula after accounting for instrumental broadening. The carbide coating porosity was measured using X-ray computed tomography using a Phoenix Nanotom X-ray nanotomograph (GE Sensing & Inspection Technologies, Wunstorf, Germany), equipped with AxioVision image analysis software (4.8.2.0). The tests were carried out on 10 areas of the coating. Examination of the surface topography of the coatings and quantification of the surface roughness parameters, specifically R_a_ (the mean deviation of the surface profile from the mean line) and R_z_ (the mean of the absolute values of the five highest peaks and five deepest valleys within a specified sampling length), were performed utilizing an Olympus LEXT OLS4100 laser confocal microscope (Hamburg, Germany). Three measurement lines of the coating’s surface roughness were used to calculate the parameters for each type of coating. Utilizing three-dimensional imaging and subsequent analytical procedures facilitated accurate delineation and characterization of the geometric structure of the examined surfaces.

### 2.3. Mechanical Properties and Scratch Resistance

**Figure 1:**
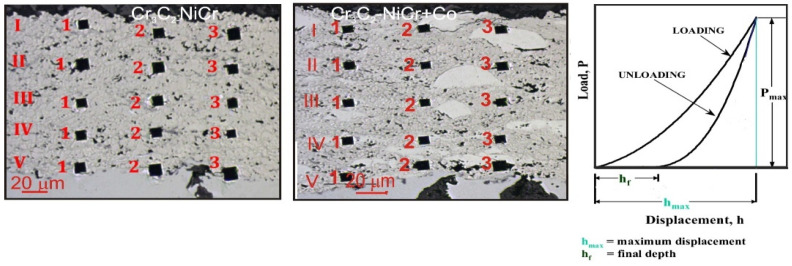
Measurement of microhardness (H_IT_) by matrix distribution on the cross-section of the coating and typical relationship between load and displacement during indentation.

Studies of the mechanical properties, which included indentation measurements of the hardness (H_IT_), Young’s modulus of elasticity (E_IT_), and fracture toughness (K_IC_), were carried out using the multifunctional measurement platform Micro Combi Tester from the Swiss company CSM Instruments. H_IT_, E_IT_, and K_IC_ were determined according to sample indentation (cross-section of coating/substrate samples) using a Vickers diamond indenter. Every cycle of loading and unloading involved continuous measurements of the indentation’s load and depth of penetration. The maximum load value for the hardness measurement and Young’s modulus was 1 N, the load and unload speed was 2 N/min, the maximum hold time was 10 s, and the contact force was 0.03 N. For The micromechanical parameters were analyzed using Oliver and Pharr’s method, which computed the penetration curve’s hardness (H_IT_) and Young’s modulus of elasticity (E_IT_) (Figure 1). For each coating/substrate system, the microhardness was measured using a matrix distribution with 15 measuring sites on the coating’s cross-section (Figure 1). The measurement positions along one measuring line, I, II, II, IV, and V, were precisely defined using the special “Visual Advanced Matrix” module thanks to the integrated light microscope.

**Figure 2:**
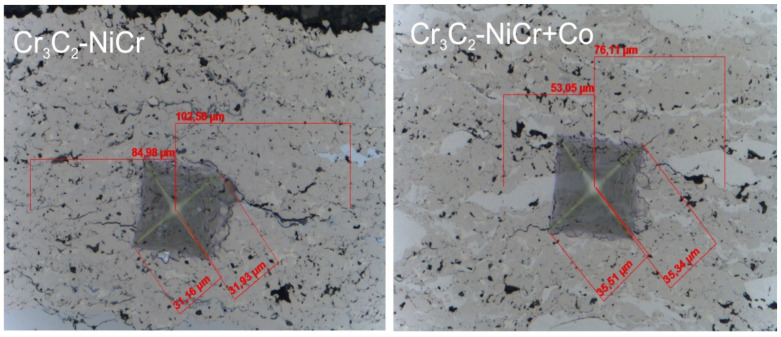
Scheme for measuring indentation fracture toughness (K_IC_) in the Cr_3_C_2_-NiCr and Cr_3_C_2_-NiCr+Co coatings at a load of 20 N.

The indentation fracture toughness, that is, the critical value of the stress intensity coefficient (K_IC_), was determined through direct measurement of the length of the cracks appearing in the corners as a result of the penetration of a Vickers indenter under the influence of specified loads: 5, 10, 15, and 20 N (the speed of loading and unloading was 40 N/min, the maximum load holding time was 10 s, and the contact load was 0.03 N). Using an integrated light microscope, the lengths of the cracks and the indentation diagonals were measured for this purpose (Figure 2). Three indentations were made in each coating/substrate type sample at a given load. After determining the total length of the cracks, the type of cracks was identified, taking into account the length ratio l/a. When the l/a ratio is > 1.5, the Anstis formula [33] is used. Two factors must be considered in order to calculate the fracture toughness: the load (P) and the crack’s length (l).

Anstis formula:

(1) $$K_{\mathrm{IC}}=0.016\;{\left(\frac{E}{HV}\right)}^{0.5}\cdot \frac{P}{c^{1.5}}$$

where K_IC_—the fracture toughness coefficient, P—the indenter load [N], HV—Vickers hardness, E—Young’s modulus of elasticity [MPa], c = a + l—the length of half of the indent’s diagonal + the length of the crack initiated from the corner of the Vickers indent [m], a—the length of half of the indent’s diagonal [µm], l—length of the crack initiated from the corner of the Vickers indent [µm].

**Figure 3:**
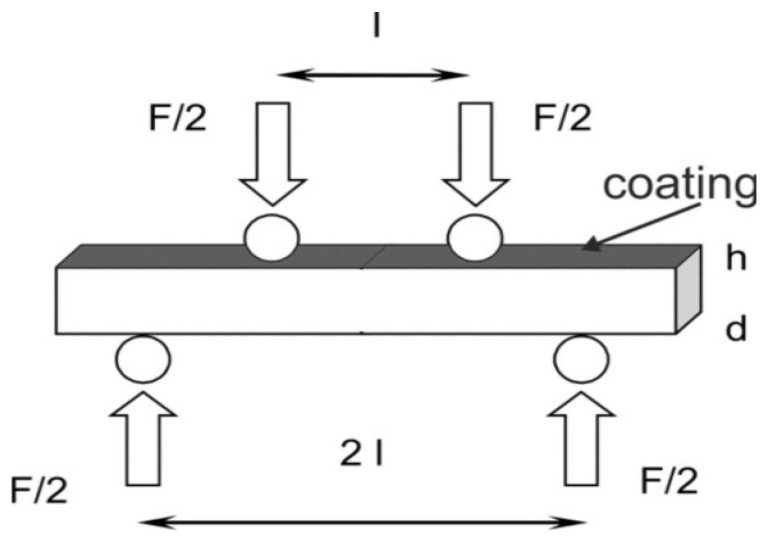
A schematic diagram of the 4-point bend test.

Using a specifically made holder for samples measuring 36 × 13 × 3 mm^3^, the Instron 8800M testing equipment (Instron, Norwood, MA, USA) was used to conduct a 4-point bending test (Figure 3) to assess the strength of the coating/substrate joint. The supports were spaced 25 mm apart, and the rate of deformation was 1 mm/min. For one test, three samples were used. Using a scanning microscope, fracture surfaces were seen following the 4-point bending test.

The bending strength was calculated according to the formula:

(2) $$\sigma =\frac{3}{2}\cdot F_{f}\frac{l}{d\cdot h^{2}}$$

where σ—the bending strength [MPa] F_f_—the load at a given point on the load deflection curve [N], l—the load spacing [mm], d—the width of the specimen [mm], h—the height of the specimen [mm].

**Figure 4:**
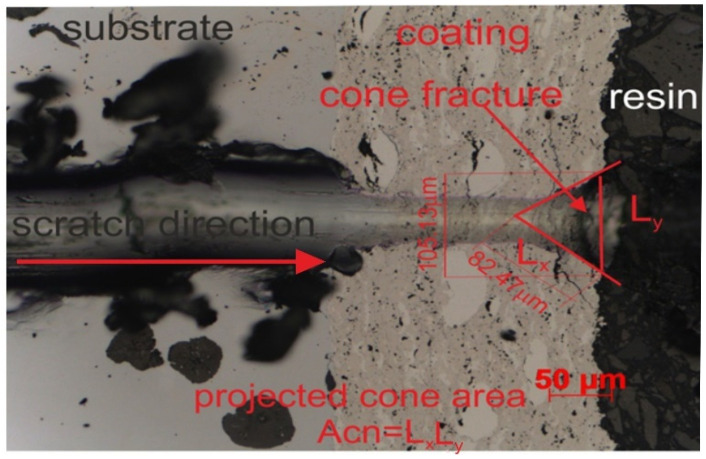
An example image of a scratch track in the substrate/composite coating/resin system created at a load of 10 N.

Tests were conducted to assess the adhesion of the coatings to the substrate and determine various mechanical types of damage, such as the depth of the penetration by the indenter, the formation of cracks, and the initiation of delamination along the scratch path. These tests were performer using a Rockwell C-type diamond indenter with a radius of curvature of 100 µm. Various penetrator forces of 5, 10, 15, 20, and 25 N were applied. The experiments utilized a multifunctional measuring platform (Micro Combi Tester, Buchs, Switzerland) equipped with Anton Paar scratch test heads, following the guidelines outlined in the standard [34]. The cross-sectioned samples were placed in DuroFast hard epoxy resin and tested. After that, they were polished according to the normal procedures for metallographic samples. When performing the scratch test, a steady stress is applied, and the indenter travels from the substrate through the coating and into the resin, encasing the sample. The scratch length was 1.2 mm. The indenter speed was 0.4 mm/min. Three scratches were made under a specified penetration load. Failure of the coating/substrate system was detected and evaluated by observing the resulting scratch on light and scanning electron microscopes. The critical load is the typical force at which failure happens. The quality of the coating-substrate bond is defined by the critical loads for cohesive and adhesive cracks, which were established. Furthermore, the projected area of a cone-shaped fracture in the coating was determined after the scratch test, Acn = L_x_·L_y_ (Figure 4), for the constant load scratch force, determining the cohesion of the coatings, and even the wear resistance was measured using a light microscope.

## 3. Results and Discussion

### 3.1. Identification of the Coating Systems’ Microstructure and Phases: Cr_3_C_2_-NiCr/Ductile Cast Iron and Cr_3_C_2_-NiCr+Co/Ductile Cast Iron

**Figure 5:**
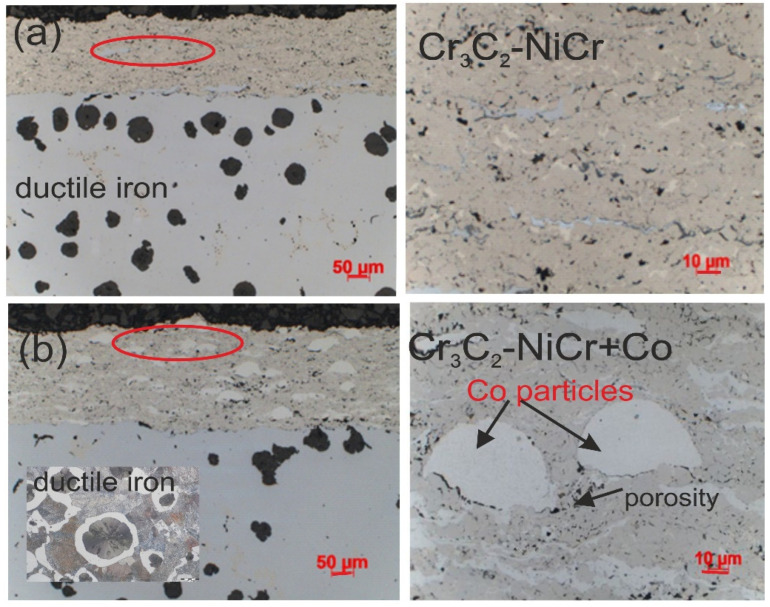
Microstructures of the systems at low and high magnification: (**a**) Cr_3_C_2_-NiCr/ductile cast iron and (**b**) Cr_3_C_2_-NiCr+Co/ductile cast iron.

**Figure 6:**
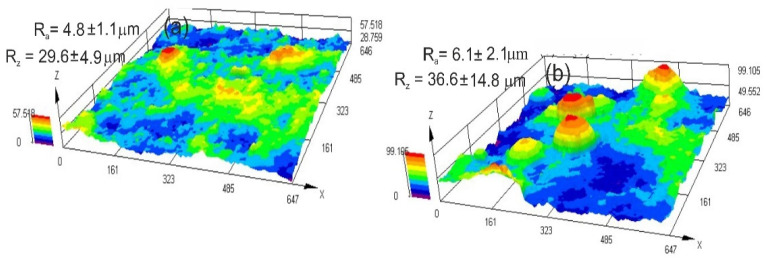
Three-dimensional view recorded using a confocal laser scanning microscope of surfaces of (**a**) Cr_3_C_2_-NiCr and (**b**) Cr_3_C_2_-NiCr+Co coatings.

The chromium carbide coatings’ microstructure, with and without metallic particles, was typical for thermal spraying; that is, layers of flattened powder particles formed the grains, which undergo geometric changes and plastic deformation during the HVOF process. Furthermore, the coatings were characterized by a compact structure without cracks. Additionally, there were few pores and oxide impurities in the coatings, and they showed good adherence to the substrate with a continuous interface. All these attributes together indicate ideal application circumstances, which, in turn, guarantee the creation of coatings of a superior quality. The NiCr alloy matrix of both coatings contained fine variously sized chromium carbide particles embedded into it, and the composite coating contained large partially melted Co particles that changed in height and length when they came into contact with the substrate (Figure 5). The porosity decreased and the surface roughness parameters increased in the Cr_3_C_2_-NiCr+Co composite coating compared to the coating without cobalt particles. The composite coating has an average porosity of 2.3 ± 0.6%. The surface roughness parameters R_a_ and R_z_ have values of 6.1 ± 2.1 µm and 36.6 ± 14.8 µm, respectively. For the Cr_3_C_2_-NiCr coating, the porosity and the values of R_a_ and R_z_ parameters are, respectively, 3.6 ± 0.8%, 4.8 ± 1.1 μm, and 26.9 ± 4.9 µm. A beneficial effect on porosity reduction is seen when ductile Co particles are added to the carbide coating. This can be explained by the way that Co particles operate as a kind of “cushion”, supporting and softening the impact of the Cr_3_C_2_-NiCr particles. This interaction facilitates densification, which is more difficult in a Cr_3_C_2_-NiCr coating because of its reduced ductility and increased hardness. But the higher roughness parameter of the composite coating may be due to the crystallization of the elongated-shaped Co particles and their “island” arrangement within the coating matrix, potentially providing better abrasion resistance. A surface image obtained from the 3D scaling of the Cr_3_C_2_-NiCr and Cr_3_C_2_-NiCr+Co coatings is shown in Figure 6.

**Figure 7:**
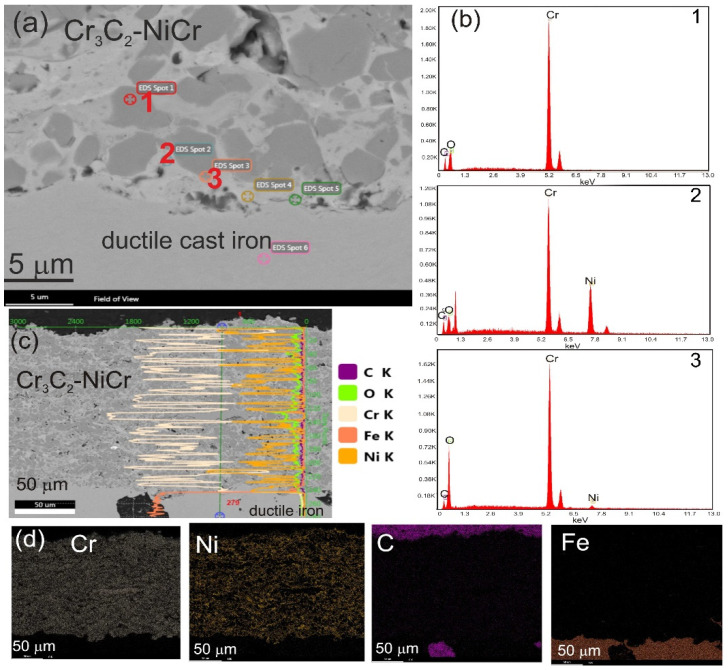
(**a**) Cross-sectional SEM images of Cr_3_C_2_-NiCr coatings; (**b**) EDS spectra taken from the marked points 1, 2, and 3; (**c**) linear representation of concentrations of C, Cr, Fe, and Ni; and (**d**) mapping the distribution of Cr, Ni, C, Fe taken from the region of interface.

**Figure 8:**
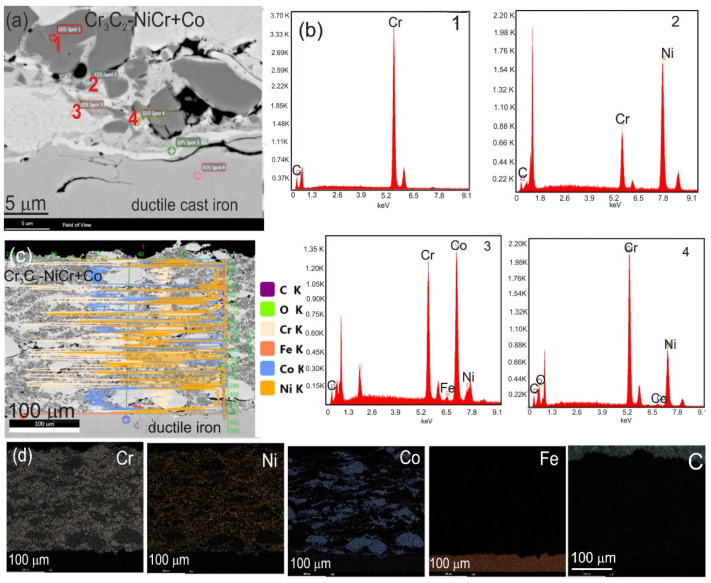
(**a**) Cross-sectional SEM images of Cr_3_C_2_-NiCr+Co coatings; (**b**) EDS spectra taken from the marked points 1, 2, 3, and 4; (**c**) linear representation of concentrations of C, Cr, Fe, Co, and Ni; and (**d**) mapping the distribution of Cr, Ni, Co, Fe, and C taken from the region of interface.

Using the SEM-EDS microanalysis, surface, linear, and point analyses of the chemical composition were carried out in order to demonstrate in detail the differences in the chemical composition of the Cr_3_C_2_-NiCr coating and the composite coating (Cr_3_C_2_-NiCr+Co) (Figure 7 and Figure 8). There are places in the coatings with different degrees of remelting (the dendritic structure is diagnostic of areas incorporating Co particles) and a notable concentration of either nickel or chromium. Although the metallic phase Ni-Cr is present in the light matrix of the composite coating (Cr_3_C_2_-NiCr+Co), the black grains have a high chromium content, indicating that they are chromium carbide grains. Generally, the studied coatings and the coating/substrate interface had chromium carbide grain sizes ranging from 0.5 to 2 µm.

The lack of elemental penetration (diffusion) from the base material to the coating and vice versa suggests that the coated material was not mechanically mixed, and the microstructure of the cast iron remained unchanged following the spraying process (ferrite and pearlite comprise the initial and post-spraying cast iron matrix, Figure 5b).

**Figure 9:**
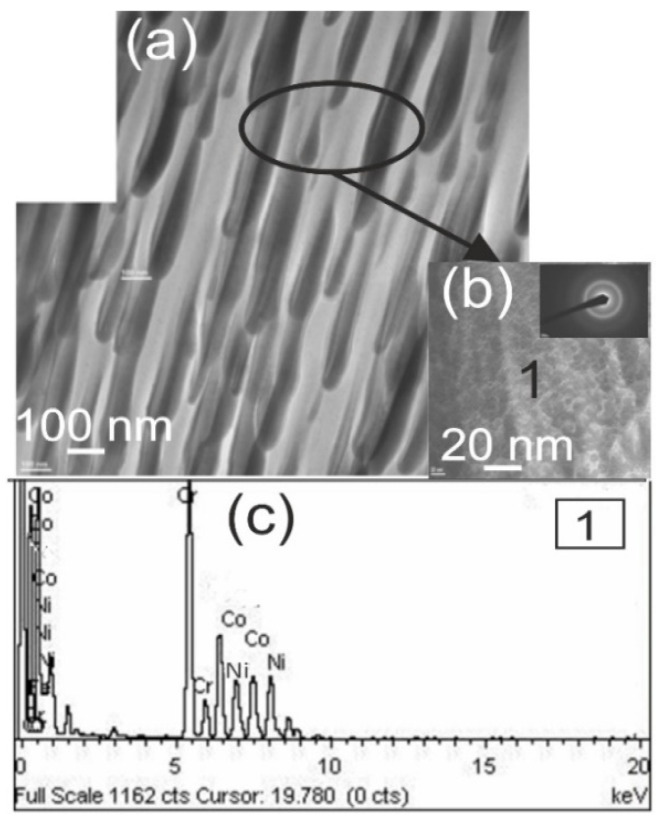
TEM analysis of the composite coating (Cr_3_C_2_-NiCr+Co) deposited on ductile cast iron: (**a**) representative TEM image; (**b**) area diffraction pattern indicating the presence of an amorphous area; and (**c**) EDS spectrum collected from the designated point.

Detailed microstructural tests of the composite coating carried out on a thin TEM foil from cross-section of the sample showed a highly fine crystalline structure with a band-like character. In the coating microstructure, there are longitudinal bands with thicknesses of 100–300 nm arranged parallel to each other. The presence of amorphous areas inside these bands was confirmed according to the diffractogram, which only showed halo rings (Figure 9). The Cr, Ni, and Co particles that make up the coating were identified using the energy-dispersive X-ray spectroscopy (EDS) technique, which also allowed us to study the chemical point composition of the coating.

**Figure 10:**
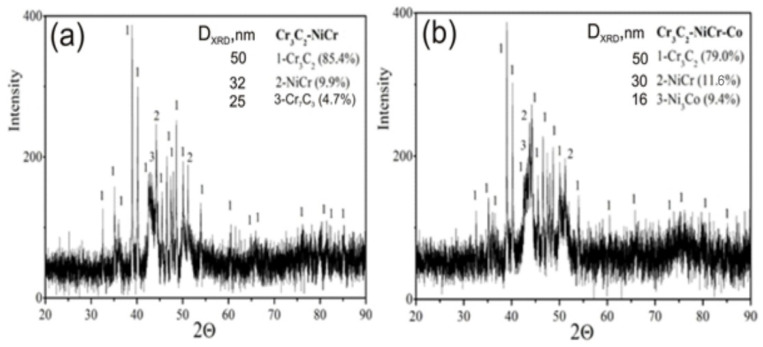
XRD patterns of the coatings: (**a**) Cr_3_C_2_-NiCr and (**b**) Cr_3_C_2_-NiCr+Co.

The phases of the carbide coatings were identified using X-ray analysis as Cr_3_C_2_, Cr_7_C_3_, NiCr, and Ni_3_Co (Figure 10). Furthermore, the weight percentage of each phase as well as the average crystallite size of each phase in the tested coating were established. The Cr_3_C_2_ phase made up 85.4% of the Cr_3_C_2_-NiCr coating, whereas the phases NiCr and Cr_7_C_3_ had corresponding amounts of 9.9% and 4.7%. The coating contains a relatively low content of Cr_7_C_3_ (resulting from the decomposition of Cr_3_C_2_ due to the impact of the spray jet on the powder grains), indicating a low degree of decomposition of the Cr_3_C_2_ carbide into Cr_7_C_3_. In the composite coating (Cr_3_C_2_-NiCr+Co), the volume fraction of the Cr_3_C_2_ phase in the coating structure is also significant (79%), which is associated with smaller losses of Cr_3_C_2_ during the coating spraying process. On the other hand, the volume fraction of the Ni_3_Co phase (9.4%) is comparable to the NiCr phase (11.6%). It is important to notice that the extremely fine crystalline structure of the coating is reflected in the average crystallite sizes of particular phases. Reducing the size of the chromium carbide shortens the mean free path in the matrix, thereby enhancing the coating’s resistance to deformation, hardness, and reducing the likelihood of binder phase extrusion. [15,21]. The presence of Cr_7_C_3_ carbide in the NiCr matrix, originating from the decomposition of Cr_3_C_2_, has the potential to alter the microstructure of the coating, thereby enhancing its resistance to cracking and wear. The Cr_7_C_3_ phase is characterized by a relatively high critical stress intensity coefficient (K_IC_) value of 2.64–4.53 MPa m^1/2^ [13]. For Cr_3_C_2_ chromium carbide produced using pulsed electric current pressure sintering, the critical value of the stress intensity coefficient (K_IC_) is 7.1 MPa m^1/2^ [35]. In addition, very fine carbide phases may have an impact on a reduction in crack propagation. It is worth mentioning that the Cr_3_C_2_ phase (HV_IT_ = 18.3 GPa [36]) is characterized by a higher microhardness in relation to the Cr_7_C_3_ phase (HV_IT_ = 16.2 GPa) [35] and a twice-higher module of elasticity (416 GPa) compared to Cr_7_C_3_ (226 GPa) [17]. Therefore, it is not anticipated that the formation of a multi-phase coating structure with a higher concentration of retained Cr_3_C_2_ and a limited number of brittle phases will have a negative effect on its anti-wear properties. Some researchers have proposed that the presence of an amorphous structure with chromium carbides, such as Cr_7_C_3_ and Cr_23_C_6_, enhances the cohesiveness of the hard Cr_3_C_2_ particles with the binder phase. As a result, the coating’s ductility and wear resistance are enhanced [24,37].

### 3.2. Co Particles’ Effects on Coating Systems’ Mechanical and Tribological Characteristics: Cr_3_C_2_-NiCr/Ductile Cast Iron and Cr_3_C_2_-NiCr+Co/Ductile Cast Iron

**Figure 11:**
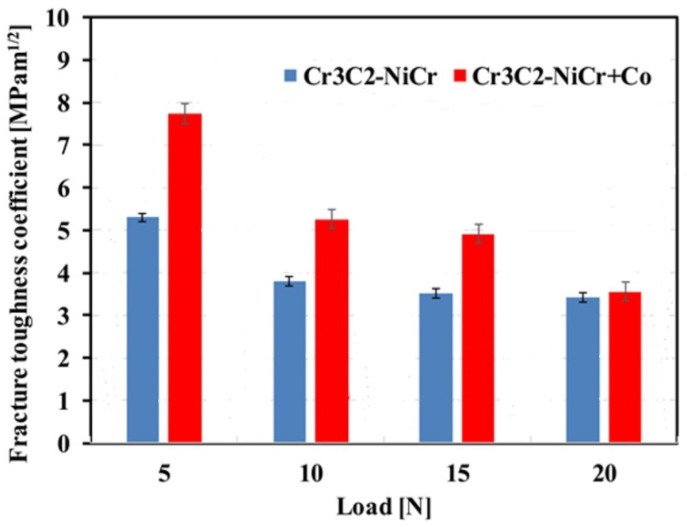
Comparison of the fracture toughness coefficient for Cr_3_C_2_-NiCr and Cr_3_C_2_-NiCr+Co coatings.

Micromechanical measurements of the coating systems’ cross-sections (Cr_3_C_2_-NiCr/ductile iron and Cr_3_C_2_-NiCr+Co/ductile iron) were analyzed, and the results showed that the addition of metallic particles significantly decreased the hardness (H_IT_) of the carbide coating (Table 2 and Table 3). In both the Cr_3_C_2_-NiCr and composite coatings (Cr_3_C_2_-NiCr+Co), the maximum microhardness was noted at a depth of around 200 μm from the surface. The improvements in the coating cohesiveness and strain hardening that occur throughout the spraying process are responsible for this result. On ductile cast iron, the microhardness of the Cr_3_C_2_-NiCr and (Cr_3_C_2_-NiCr+Co) coatings is differentiated; at a depth of 25 μm from the surface, it is 9.72 ± 2.21 and 7.97 ± 0.76 GPa; at a depth of 200 μm, it is 12.46 ± 2.46 and 10.09 ± 0.51 GPa, respectively; and it then decreases to a value of approximately 7.80 ± 1.65 GPa near the substrate. Additionally, the maximum value of Young’s modulus is found for the carbide coating with and without metal particles at a depth of 200 μm. It is significant to notice that the coating free of metal particles has a higher hardness-to-Young’s-modulus ratio than the composite coating. The material’s increased ability to withstand strains within an elastic deformation regime without plasticizing is indicated by this lower ratio. Compared to the coating free of metal particles, the composite coating notably shows a lower value for the ratio of hardness to its Young’s modulus (H^3^/E^2^). This shows that there is greater resistance to plastic deformation during indentation in the composite covering. This indicates a more favorable reaction to localized stresses and gives information about the material’s behavior under concentrated loads. This suggests that in addition to having a better resistance to plastic deformation, the material also has a higher degree of elasticity, which can help to reduce some types of wear and failure. The Cr_3_C_2_-NiCr coating’s brittleness is decreased by a localized decrease in hardness brought on by the doping of the metal particles. The softer Co particles added to the brittle chromium carbide grains improve the coating’s plastic deformation flexibility. Because of this combined action, coatings are produced that have a healthy balance between high hardness and flexibility, making them resistant to fatigue wear and cracking. For the composite coating in the load range of 5–20 N, there are higher critical fracture toughness coefficient (K_IC_) values than for the coating without metallic particles (Figure 11). An important characteristic of supersonic sprayed coatings is that the initiated cracks in the coating/substrate system’s cross-section propagate in a direction parallel to the coating/substrate interface [22]. For the composite coating, the average K_IC_ values in the load range of 5–20 N are between 7.74 and 3.56 MPa m^1/2^, and for the coating free of metallic particles, they are between 5.30 and 3.42 MPa m^1/2^. Due to the coating’s heterogeneous structure, tests of the indentation fracture toughness (K_IC_) of the composite coating are characterized by a wider dispersion of results. The greater plastic deformation capacity of the composite coatings than the Cr_3_C_2_-NiCr coating is indicated by higher K_IC_ values for the composite coatings in the load range of 5–20 N, shorter cracks, and a higher value in the E/H ratio (the coating becomes more plastic, and cracking is constrained by the added metallic particles). The NiCr alloy matrix of the coating is characterized by a higher absorption energy after the addition of metallic particles, which reduces the growth and propagation of cracks, improving its fracture toughness and also indicating better cohesion between the lamellae in the supersonic sprayed coating. Additionally, the lower porosity of the composite coatings may lead to higher K_IC_ values.

**Figure 12:**
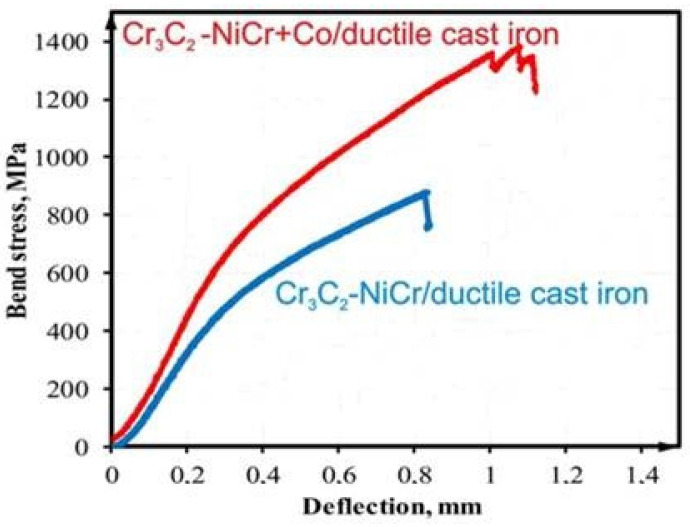
Bend test curves recorded for systems: Cr_3_C_2_-NiCr/ductile cast iron and (Cr_3_C_2_-NiCr+Co)/ductile cast iron.

Figure 12 compares the bending test results, with respect to the bending stress–deflection value, for the systems Cr_3_C_2_-NiCr/ductile iron and (Cr_3_C_2_-NiCr+Co)/ductile iron. The maximum bending stress of the Cr_3_C_2_-NiCr+Co/ductile cast iron system increased by more than 1.5 times when compared to the Cr_3_C_2_-NiCr/ductile cast iron system. The highest bending stresses for the (Cr_3_C_2_-NiCr+Co)/ductile cast iron and Cr_3_C_2_-NiCr/ductile cast iron systems are 880 ± 12 MPa and 1330 ± 15 MPa, respectively. The deflection is 0.83 mm for the Cr_3_C_2_-NiCr/ductile cast iron system and 1.12 mm for the composite coating system. These values indicate a deflection followed by a decrease in stress, leading to system failure. When comparing the resulting curves, it can be observed that the system with the coating free of metallic particles experiences a 66% decrease in deflection (reducing to a value of 0.83 mm) and a decrease in the strength parameters of the bending process. In the absence of Co particles, chromium carbide coatings become more brittle and tougher, reducing the amount of energy that can be released during plastic deformation. Crack propagation is accelerated, and the deflection range is reduced due to the rapidly increasing load.

**Figure 13:**
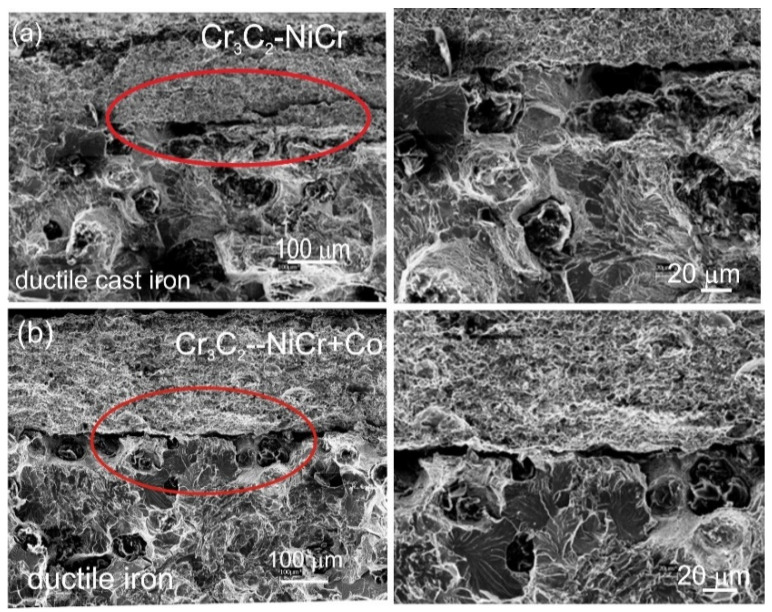
Scanning micrographs of the fracture surface of the systems: (**a**) (Cr_3_C_2_-NiCr)/ductile cast iron and (**b**) (Cr_3_C_2_-NiCr+Co)/ductile cast iron after bend test.

Interestingly, despite differences in the linear expansion coefficients between the substrate and coating, there was no significant loss in strength in any of the studied coating systems due to internal stresses. The 75Cr_3_C_2_-25NiCr coating, with a coefficient of thermal expansion of 11.10 × 10^−6^ K^−1^ [7], similar to that of the iron-based substrate (13.2 × 10^−6^ K^−1^), does not delaminate from the substrate, indicating minimal internal stresses during spraying, as the coefficients of linear expansion for both the substrate and coating are very close. Furthermore, there should not be any internal stresses that could compromise the mechanical durability of the substrate–coating bond because the substrate’s and coating’s coefficients of linear expansion, denoted as Co (12 × 10^−6^ K^−1^), are extremely close to each other during the spraying process. Additionally, the (Cr_3_C_2_-NiCr+Co)/ductile cast iron system is stronger because fewer stresses are created at the coating–substrate interface. This is due to the composite coating’s elasticity modulus being less different from the substrate’s modulus (E = 165 GPa for ductile cast iron) than that of the coatings without metallic particles is [38]. The sample fractures observed under a scanning electron microscope following the bending tests (Figure 13) demonstrate that, in the Cr_3_C_2_-NiCr/ductile cast iron system, degradation occurs both along the coating–substrate interface and within the coating, whereas in the composite coating system, it only happens along the interface.

**Figure 14:**
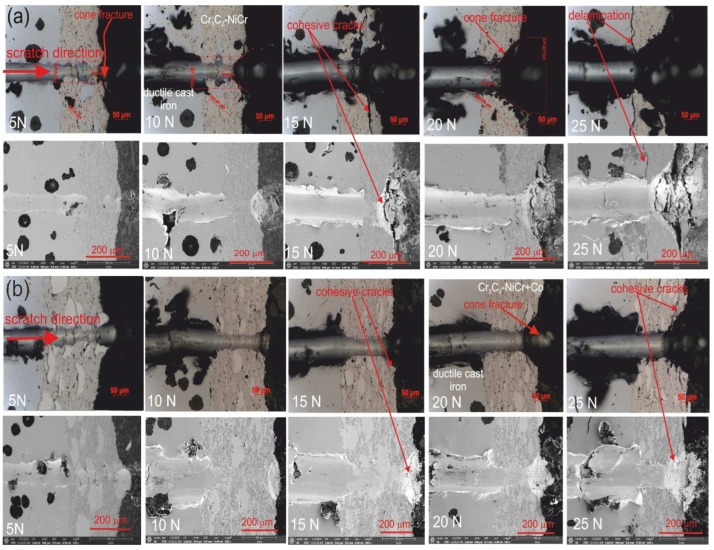
LM/SEM micrographs showing cone-shaped fracture occurring during the scratch bond strength test for systems: (**a**) Cr_3_C_2_-NiCr/ductile cast iron and (**b**) Cr_3_C_2_-NiCr+Co/ductile cast iron.

**Figure 15:**
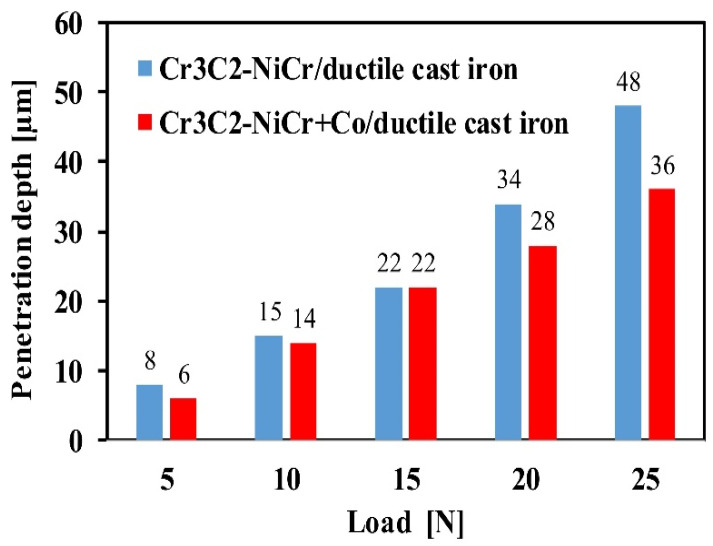
Comparison of the penetration depth of the indenter for Cr_3_C_2_-NiCr/ductile cast iron and Cr_3_C_2_-NiCr+Co/ductile cast iron systems during the scratch test.

The results of the scratch test, carried out on the coating/substrate systems’ cross-section with constant loads of 5, 10, 15, and 20 N, are displayed in Figure 14 and Table 4 and Table 5. The projected area cone (Acn) values of the composite coating system are generally lower than those of the Cr_3_C_2_-NiCr/ductile cast iron system, suggesting that the (Cr_3_C_2_-NiCr+Co)/ductile cast iron system possesses a stronger scratch bond strength. Both coating systems exhibit an interior cone-shaped fracture, indicating cohesive failure within the coating/substrate system. Cohesive cracks initiate simultaneously in the Cr_3_C_2_-NiCr coating at a relatively low load of 5 N, while in the composite coating, they begin at 10 N. At the highest load of 25 N, significant fractures occur in the Cr_3_C_2_-NiCr coating surrounding the scratch, leading to delamination of the coating from the substrate and adhesive degradation. Since the composite coating (Cr_3_C_2_-NiCr+Co) has good adhesion to the substrate at very high loads above 20 N (only cohesive cracks form), such catastrophic destruction is not observed for this coating. The addition of cobalt particles to the coating increases both its ductility and its resistance to scratching. Specifically, above a contact stress of 15 N, it was demonstrated that the coating’s metallic particles limited the intender’s penetration depth, making scratching more difficult (Figure 15). Importantly, the addition of metallic particles to the chromium carbide powder deflects and prevents microcracks, improving the coating’s adhesion to the substrate.

## 4. Conclusions

The key findings are summarized as follows:

The composite coating (Cr_3_C_2_-NiCr+Co) applied by means of the HVOF technique to ductile cast iron is characterized by low porosity, a compact lamellar structure, and good adhesion to the substrate. The coating’s microstructure contains relatively large partially melted Co particles as well as highly fine chromium carbide particles embedded into nickel–chromium.

The hardness of the composite coating (Cr_3_C_2_-NiCr+Co) is lower than that of the Cr_3_C_2_-NiCr coatings. This is mainly attributed to the distribution of Co particles within the matrix and a decrease in the concentration of the hard carbide phases.

Cobalt was added to chromium-carbide-based coatings to improve their mechanical properties, such as plasticity and fracture toughness. This enhancement is attributed to cobalt’s influence on the binding phase, which alters the cohesion and the coating’s ability to transfer loads efficiently.

The (Cr_3_C_2_-NiCr+Co)/ductile cast iron system demonstrates an improved bending strength and crack resistance, with failures primarily occurring at the coating–substrate interface due to the high quality and ductility of the coating. In contrast, failures in the Cr_3_C_2_-NiCr/ductile cast iron system are experienced within the coating and along the interface.

This effectively indicates that the (Cr_3_C_2_-NiCr+Co)/ductile cast iron system demonstrates stronger bonding compared to using only Cr_3_C_2_-NiCr on the same substrate. This improvement occurs because Co particles can inhibit or deflect crack propagation within their distribution regions, thereby strengthening the bond between the coating and substrate.

## Figures and Tables

**Table 1:** Spraying parameters of Cr_3_C_2_-NiCr coatings using HVOF technology.

Gun Movement Speed, mm/s	Oxygen, L/min	Kerosene, L/h	Powder Feed Rate, g/min	Powder Feed Gas, L/min	Spraying Distance, mm
583	850	24	65	Nitrogen, 9.5	370

**Table 2:** Indentation hardness (H_IT_) and Young’s modulus (E_IT_) values of Cr_3_C_2_-NiCr coating.

Indenter Print Image	Measuring Line	H_IT_ [GPa]	E_IT_ [GPa]	Average H_IT_ [GPa]	Average E_IT_ [GPa]
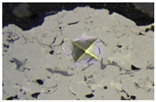	I	12.53	189.6	9.72 ± 2.21	179.35 ± 2.34
		9.47	178.88		
		7.16	169.50		
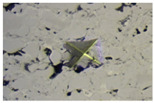	II	8.35	196.41	9.34 ± 1.36	202.60 ± 1.97
		8.43	195.53		
		11.23	215.87		
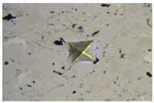	III	11.85	225.03	11.37 ± 0.73	216.95 ± 12.56
		10.32	194.43		
		11.94	231.39		
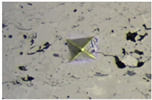	IV	10.46	207.91	12.46 ± 2.46	223.18 ± 10.47
		11.09	220.64		
		15.83	240.99		
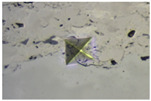	V	10.05	205.21	8.04 ± 1.63	196.75 ± 5.37
		7.87	205.21		
		6.19	179.82		

**Table 3:** Indentation hardness (H_IT_) and Young’s modulus (E_IT_) values of Cr_3_C_2_-NiCr+Co coating.

Indenter Print Image	Measuring Line	H_IT_ [GPa]	E_IT_ [GPa]	Average H_IT_ [GPa]	Average E_IT_ [GPa]
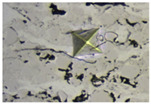	I	8.78	169.29	7.97 ± 0.76	168.26 ± 1.84
		7.85	164.36		
		7.28	171.14		
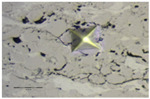	II	9.67	200.82	9.76 ± 1.63	204.68 ± 6.58
		11.44	220.40		
		8.18	192.83		
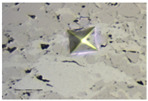	III	9.84	212.38	10.15 ± 1.13	209.71 ± 1.93
		11.40	211.19		
		9.21	205.55		
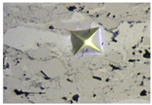	IV	9.92	211.50	10.09 ± 0.51	218.12 ± 4.33
		10.67	228.76		
		9.69	214.10		
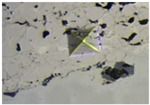	V	8.45	169.50	7.63 ± 1.67	184.63 ± 11.67
		8.62	209.56		
		5.82	174.83		

**Table 4:** Average results from scratch bond tests for the investigated coatings.

Coating System	Load [N]	Lx [µm]	Ly [µm]	A_cn_ × 10^−3^ [mm^2^]
Cr_3_C_2_-NiCr/ductile cast iron	5	56.16	71.76	4.03
	10	103.37	104.41	10.79
	15	200.00	212.50	42.50
	20	206.79	242.28	50.10
	25	delamination		
Cr_3_C_2_-NiCr+Co/ductile cast iron	5	51.34	76.33	3.92
	10	83.43	104.17	8.69
	15	111.36	137.50	15.31
	20	141.67	191.65	27.15
	25	166.67	316.67	52.78

**Table 5:** Percentage of the characteristic forms of failure under constant load (no cracks, cohesive cracks, adhesive cracks) according to the standard ISO 27307:2015 “Thermal spraying, Evaluation of adhesion/cohesion of thermal sprayed ceramic coatings by transverse scratch testing” [39].

Coating System	Load [N]	No Crack [%]	Cohesive Crack [%]	Adhesive Crack [%]	Maximum Load at Which Cohesive Cracks Appears	Maximum Load at Which Adhesive Cracks Appears
Cr_3_C_2_-NiCr/ductile cast iron	5	85	15	0	over 5 N	
	10	70	30	0		
	15	50	50	0		
	20	70	30	0		
	25	0	0	100		delamination
Cr_3_C_2_-NiCr+Co/ductile cast iron	5	95	5	0		
	10	90	10	0	over 10 N	
	15	80	20	0		
	20	70	30	0		
	25	60	40	0		

## Data Availability

Data are contained within the article.
